# Concomitant knockout of target and transporter genes in filamentous fungi by genome co‐editing

**DOI:** 10.1002/mbo3.1280

**Published:** 2022-04-13

**Authors:** Koichi Tamano

**Affiliations:** ^1^ Bioproduction Research Institute National Institute of Advanced Industrial Science and Technology (AIST) Sapporo Hokkaido Japan; ^2^ AIST‐Waseda University Computational Bio Big‐Data Open Innovation Laboratory (CBBD‐OIL), AIST Waseda University Tokyo Japan

**Keywords:** autotrophy, breeding, filamentous fungi, genome co‐editing, non‐genetically modified

## Abstract

In most countries, genetically modified microorganisms are not approved for use for fermentation in the food industry. Therefore, random mutagenesis and subsequent screening are performed to improve the productivities of valuable metabolites and enzymes as well as other specific functions in an industrial microbial strain. In addition, targeted gene knockout is performed by genetic recombination using its enzyme genes as selectable markers to maintain self‐cloning status. However, random mutagenesis has a drawback as it does not guarantee improvement of the targeted function. Conversely, self‐cloning is rarely used to breed an industrial microbial strain. This is probably because a self‐cloning strain is similar to a genetically modified strain, as both undergo homologous recombination, although exogenous genes are not introduced. In this article, I discuss the usefulness of genome editing technology as a substitute for conventional techniques to breed filamentous fungal strains. This article particularly focusses on “genome co‐editing,” a genome editing technology used for knocking out two genes concomitantly, as reported in *Magnaporthe grisea* and *Aspergillus oryzae*. Especially, when genome co‐editing is applied to a target gene and a membrane transporter gene that aid the entry of toxic compounds into cells, the resulting clone can be categorized as an autotrophic and non‐genetically modified clone. Such a clone should easily apply to industrial fermentation without being restricted by a genetically modified status. Genome co‐editing will also be used to construct mutant strains with multiple target gene knockouts by eliminating multiple membrane transporter genes. This could substantially improve the productivities of valuable metabolites and enzymes in a stepwise manner. Thus, genome co‐editing is considered a potentially powerful method to knockout single or multiple target genes that can contribute to the breeding of filamentous fungal strains in the food industry.

## Graphical abstract

Illustrative explanation of the key difference between genome editing of the target gene alone and genome co‐editing of both target and membrane transporter genes. Genome editing generated colonies all around the plate due to no selection pressure, while genome co‐editing generated single colonies due to selection pressure. Genome co‐editing can generate a knockout mutant of the target gene in both autotrophic and non‐genetically modified forms.

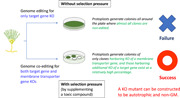

## 1 INTRODUCTION

In countries where genetically modified (GM) strains have not been approved for use for fermentation in the food industry, microbial strains are bred by random mutagenesis or mating, followed by screening for the improved strains (Deckers et al., 2020; Mahaffey et al., 2016). However, random mutagenesis requires labor‐intensive screening of a large number of mutant strains and does not always generate mutants with improved targeted function. Particularly, because they form giant colonies, it is much more difficult to perform high‐throughput screening on agar plates for filamentous fungi than for bacteria and yeasts. Moreover, one of the limitations associated with random mutagenesis is that mutations are mostly introduced in several loci of the genome and can accordingly cause an undesirable shift in growth and other phenotypes (Park et al., 2008). On the other hand, the sexual life cycle needs to be confirmed to breed microbial strains by mating; however, this has not been confirmed in some percentages of filamentous fungal species. Thus, there are multiple limitations for efficiently improving filamentous fungal strains used in the food industry. The year 2004 was the turning point for techniques for creating improved strains of filamentous fungi. Molecular factors involved in the nonhomologous end‐joining (NHEJ) pathway have been identified in *Neurospora crassa* (Ninomiya et al., 2004). Subsequent studies have identified that genes *ku70*, *ku80*, and *ligD* encode the factors that are conserved among many filamentous fungal species (Ishibashi et al., 2006; Takahashi et al., 2006). If a knockout mutant of one of the three genes is obtained by a low efficient homologous recombination (HR), it then becomes possible to construct mutant strains of targeted genes by HR with high efficiency due to loss of NHEJ capability. However, to construct recombinant mutant strains, selectable markers need to be available before constructing the strain. For this purpose, spontaneous mutant strains that are resistant to toxic compounds such as 5‐fluoroorotic acid, chlorate, and selenate are acquired, and then clones that have mutations in the enzyme genes of orotidine‐5′‐phosphate decarboxylase (de Ruiter‐Jacobs et al., 1989), nitrate reductase (Unkles et al., 1989), and ATP sulfurylase (Yamada et al., 1997), respectively, are selected by sequencing their coding regions. Subsequently, the original enzyme genes can be used as the selectable markers to construct self‐cloning gene knockout strains. The resulting strains are suitable for use in the food industry since they are not categorized as GM strains but self‐cloning strains. However, the possibility of a mutation spontaneously occurring in these enzyme genes is unknown. Moreover, there is a possibility that mutations in other genes may similarly induce resistance to toxic compounds. Thus, it is not certain that target gene knockout strains can be obtained consequently as self‐cloning forms via HR.

Recently, a novel technology called genome editing was developed. There are mainly three types of genome editing techniques: zinc finger nucleases (ZFN), TALEN, and CRISPR/Cas9 (Gupta et al., 2019). Among them, CRISPR/Cas9 seems to be the most preferred genome editing technique as it is easy to perform (Jiang et al., 2021). A single guide RNA (sgRNA) and Cas9 recombinase are the two tools required for genome editing by CRISPR/Cas9. The sgRNA can be tailor‐made by a certain biochemical company, while the Cas9 recombinase is distributed by the same company, that is, both can be obtained from the company. If a filamentous fungal strain can be transformed by the protoplast‐PEG method, a gene knockout mutant can be created by genome editing using a complex of sgRNA and Cas9 called ribonucleoprotein (RNP) in place of donor DNA. Therefore, it is important to establish the protoplast‐PEG transformation system before attempting CRISPR/Cas9 genome editing with RNP in a filamentous fungal strain.

Furthermore, it is possible to knockout two genes concomitantly at a single transformation using two varieties of RNPs. This concomitant knockout of two genes is named “genome co‐editing” (Foster et al., 2018; Todokoro et al., 2020). Genome co‐editing potentially contributes to the generation of multiple autotrophic gene knockout mutants of filamentous fungi that are nongenetically modified (non‐GM). In this article, I introduce the concept of genome co‐editing and discuss its potential application in creating autotrophic non‐GM mutants by concomitant knockout of a target gene and a membrane transporter gene.

## 2 ARTICLES REPORTING GENOME CO‐EDITING IN FILAMENTOUS FUNGI

To date, there seem to be only two articles on genome co‐editing in filamentous fungi (Foster et al., 2018; Todokoro et al., 2020). I will be discussing both articles in this section.

### 2.1 Genome co‐editing of gene pairs *SDI1* and *ALB1* or *ILV2* and *TUB2* in *Magnaporthe grisea*

Foster et al. (2018) have discovered the concomitant knockout of two genes at one transformation by genome editing. They validated the feasibility of this concept and termed it gene co‐editing. Since the two genes are knocked out in the same genome, this concept should also be called genome co‐editing. This seems to be the first study on genome co‐editing in filamentous fungi. The authors knocked out pairs of genes: *SDI1*/*ALB1* and *ILV2*/*TUB2* in *M. grisea* by genome co‐editing. Carboxin was used to select *SDI1* knockout mutants, whereas sulfonylurea was used to select *ILV2* knockout mutants. While *SDI1* encodes succinate dehydrogenase, *ILV2* encodes acetolactate synthase. These enzymes convert carboxin and sulfonylurea to toxic compounds in cells, respectively. Thus, the knockout mutants of these enzyme genes can be selected by use of these compounds. Among the knockout mutants, 0.5%–2% were reported to have undergone RNP targeting that knocked out another gene, either *ALB1* or *TUB2*, in the *SDI1* or *ILV2* knockout mutants, respectively.

### 2.2 Genome co‐editing of *thiI* and *wA* genes in *Aspergillus oryzae*

Todokoro et al. (2020) screened mutant clones of *A. oryzae* resistant to pyrithiamine, a toxic analog of thiamine, after ultraviolet‐irradiation of the wild‐type RIB40 strain spores. They isolated ten clones and sequenced their genomes; nine out of the 10 clones exhibited mutations in *thiA*, which is involved in the biosynthesis of thiamine, an essential primary metabolite. The results obtained from the nine clones coincided with the observations of a previous study (Kubodera et al., 2000). However, the remaining single clone contained an intact *thiA* sequence. This indicated that a null mutation in a different gene, and not *thiA*, conferred pyrithiamine resistance. Subsequently, they analyzed the point mutations sites in the genome of this clone and found a mutation in a membrane transporter gene. They named the gene *thiI*. It was considered that *thiI* encoded a membrane transporter of thiamine, based on homology search results. When thiamine is present in culture media, such as complete media, *A. oryzae* can utilize it for growth. On the other hand, when thiamine is absent in media, such as minimal media, *A. oryzae* can also synthesize it *de novo* for growth via its metabolism. Therefore, introducing a null mutation into *thiI* probably inhibited the incorporation of pyrithiamine into cells, whereas thiamine could still be synthesized in the mutant cells. Consequently, *thiI* null mutants could grow normally even in the presence of pyrithiamine in the medium.

Furthermore, Todokoro et al. (2020) subsequently performed genome co‐editing in *A. oryzae*. They chose the genes *wA* and *thiI* as knockout targets. They also used the CRISPR/Cas9 system for genome co‐editing and introduced a mixture of two types of RNPs, targeting *wA* and *thiI*, into *A. oryzae* RIB40 protoplasts by the protoplast‐PEG transformation method. Transformants were selected from a minimal agar medium supplemented with pyrithiamine, indicating that all transformants harbored a null mutation in *thiI*. Among the transformants, 5.5% also had a null mutation in *wA*; these transformants formed whitish colonies as they were incapable of spore pigment biosynthesis due to loss of function of *wA* (Watanabe et al., 1999).

### 2.3 Importance of genome co‐editing in obtaining knockout mutants of a target gene in filamentous fungi

**Figure 1:**
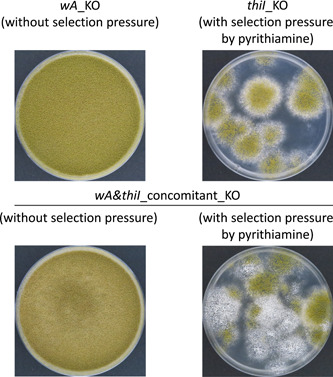
Regeneration of *Aspergillus oryzae* from protoplasts that were genome‐edited or genome‐co‐edited for *wA* and *thiI* knockouts (KOs) with or without selection pressure. Protoplasts of *A. oryzae* wild‐type strain RIB40 transformed with ribonucleoprotein (RNPs) for *wA* and/or *thiI* KO were regenerated in the presence or absence of pyrithiamine for 6 days at 30°C. Experimental parameters and conditions were in accordance with those of a previous article (Todokoro et al., 2020), except that the target sequence of single guide RNA (sgRNA) used for *wA* KO was 5′‐GATCCACTATGCTCGTAAAC‐3′, while that for *thiI* KO was 5′‐GGCGAAGACGAGACGCGAGG‐3′. Plates are depicted as follows: *wA*_KO plate without selection pressure (top left), *thiI*_KO plate with selection pressure (top right), *wA* and *thiI*_concomitant_KO plates without (bottom left) or with (bottom right) selection pressure

Interestingly, in both studies (Subsections 2.1 and 2.2), a gene whose knockout caused resistance to toxic compounds was knocked out concomitantly with the gene of interest. Here, I would like to explain the reason by presenting a study involving concomitant knockout of *wA* and *thiI* by genome co‐editing. It is natural to presume that only knocking out *wA* should suffice if the purpose of a study is to generate a *wA* knockout mutant, that is, knockout of another gene should not be required for the desired purpose. However, only attempting the knockout of *wA* by genome editing can make it difficult to obtain a knockout mutant because of an inconvenient construction system. To explain this inconvenience, I performed genome co‐editing of *wA* and *thiI* under several conditions as shown below. The experimental protocols followed those of the previous article (Todokoro et al., 2020) except that I designed new sgRNAs for *wA* and *thiI* knockouts (Figure 1).

I used 2.6 × 10^7^ protoplasts for transformation per sample in the genome editing or co‐editing process. Five plates were prepared for each transformation. Upon knocking out only *wA*, no supplementation of pyrithiamine in the agar medium resulted in cell growth in the whole plate (top left in Figure 1). On the other hand, pyrithiamine supplementation to the agar medium for selection of transformants with a concomitant knockout of *wA* and *thiI* resulted in the generation of 94 single colonies of transformants (approximately 20 colonies per plate). In addition, 39 of these colonies (41%) were whitish in appearance, indicating that they harbored both *wA* and *thiI* knockouts (bottom right in Figure 1, Table 1). Notably, the absence of pyrithiamine in this concomitant knockout transformation also led to cell growth in the whole plate (bottom left in Figure 1), similar to that under a single knockout of *wA*.

**Table 1:** Statistical data on colonies generated by genome editing or co‐editing in *Aspergillus oryzae*

Targeted gene(s)	Number of protoplasts used	Green colonies	White colonies	*wA* knockout frequency (%)	Technique used	Selection pressure	Corresponding panels in Figure 1
*wA* a	520	104	0	N.D.	Genome editing	None	Top left
*wA* and *thiI*	2.6 × 10^7^	55	39	41%	Genome co‐editing	Pyrithiamine	Bottom right

Abbreviation: N.D., not detected.

^a^ In case of single knockout of *wA* by genome editing, protoplasts with ribonucleoprotein were diluted for colony counting and then plated.

In the case of only *wA* knockout by genome editing (top left in Figure 1), colonies were generated from all protoplasts used. As a result, the plates had an intermingled and overgrown appearance. Such growth could have obscured the detection of knockout mutants due to the lack of single colonies. I thus diluted protoplasts after RNP was added for *wA* knockout, and then plated thirteen protoplasts on each plate. This was because *A. oryzae* had to be grown as giant colonies (about 2 cm in diameter) to form mature spores with detectable color (green where *wA* was intact, or white where *wA* was knocked out). Forty plates were used for this experiment. At the end of the experiment, 104 single colonies were generated from 520 protoplasts; however, they were all greenish (Table 1). This indicated that the efficiency of *wA* knockout was less than 1%. The assumption was that if more colonies were screened, a whitish colony would be found; however, it is considered that attempting to obtain *wA* knockout mutant in this manner is inefficient.

**Figure 2:**
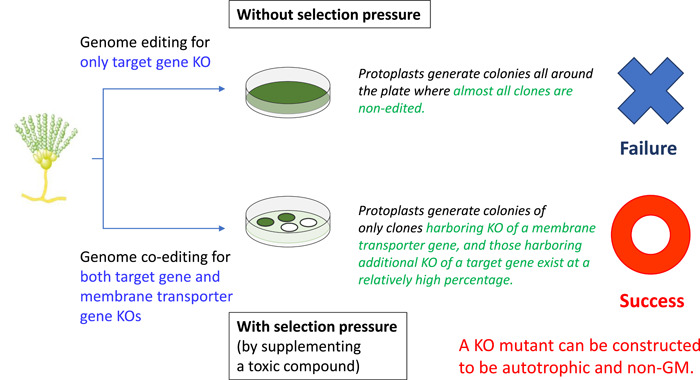
Illustrative explanation of the key difference between genome editing of the target gene alone and genome co‐editing of both target and membrane transporter genes. Genome editing generated colonies all around the plate due to no selection pressure (upper side), while genome co‐editing generated single colonies due to selection pressure (lower side). Genome co‐editing is only practically able to generate a knockout (KO) mutant of the target gene in both autotrophic and non‐genetically modified (non‐GM) forms

Figure 2 provides an illustrative explanation of how selection pressure brings about these observations. Since selection pressure did not exist under a single knockout of *wA* due to the absence of pyrithiamine, protoplasts used for transformation resulted in cell growth in the whole plate. Moreover, since all colonies were greenish, I considered that protoplasts harboring *wA* knockout were either completely or mostly absent (upper side in Figure 2). This means that the possibility of obtaining a gene knockout strain by genome editing without selection pressure is extremely low. Such a low possibility should result from a combination of the efficiency of RNP incorporation into protoplasts and the possibility of error in repairing DNA double‐strand breaks.

For reference, I also knocked out only *thiI* by genome editing. Consequently, about 20 colonies of *thiI* knockout mutants were obtained per plate under the selection pressure of pyrithiamine (top right in Figure 1). Thus, most protoplasts were not subjected to *thiI* gene knockout by genome editing and retained their original phenotypes. Moreover, as mentioned in the bottom right of Figure 1, knocking out both *thiI* and *wA* by genome co‐editing generated about 20 colonies per plate in the presence of pyrithiamine, and 41% of them appeared to be whitish (lower side in Figure 2). The white colonies indicated that the strains were knockout mutants of both *wA* and *thiI*. This means that both types of RNPs, targeting *wA* and *thiI*, were efficiently incorporated into the highly permeable protoplasts and generated concomitant gene knockout mutants at a high possibility of 41%. Conversely, neither *thiI*‐targeting RNP nor *wA*‐targeting RNP was incorporated into protoplasts that were not permeable of RNPs. Thus, it can be deduced that protoplasts are like competent cells of bacteria and have broad diversity in the efficiency of RNP incorporation. While some protoplasts have a high capacity for RNP incorporation, others have a low capacity. In addition, protoplasts with a low capacity for RNP incorporation seemed to exist at an overwhelmingly high ratio.

Regarding the gene knockout efficiency by genome co‐editing, it was 41% in the case of *wA* knockout in this study. On the other hand, there are several articles reporting marker‐free gene knockout by genome editing in filamentous fungi, such as *A. nidulans* (Nødvig et al., 2018), *A. niger* (van Leeuwe et al., 2019), and *A. oryzae* (Katayama et al., 2019). In these cases, plasmid DNAs encoding Cas9 and sgRNA in combination with or without repair DNA fragments were introduced into cells to cause knockout of targeted genes. Gene knockout efficiencies were reportedly over 50%, respectively. Thus, gene knockout efficiency by genome co‐editing, which was evaluated in this study, is considered to be almost equivalent to that by conventional marker‐free genome editing using DNA.

## 3 GENOME CO‐EDITING FOR CONCOMITANT KNOCKOUT OF TARGET AND MEMBRANE TRANSPORTER GENES: BREEDING FILAMENTOUS FUNGI RETAINING AUTOTROPHY AND NON‐GM STATUS

Concomitant knockout mutants of target and membrane transporter genes could be constructed efficiently in *A. oryzae* by genome co‐editing, as described in Subsections 2.2 and 2.3. The mutants were only subjected to concomitant gene knockout based on the repair error of DNA double‐strand break sites by the CRISPR/Cas9 system. They were not subjected to the introduction of exogenous genes throughout the whole process of mutant construction and therefore, were not categorized as GM microorganisms. Moreover, they were autotrophic and could grow normally in a minimal medium. No phenotypic change from the parental wild‐type strain was observed. Such a knockout method can be used for breeding industrial strains of filamentous fungi. The strains bred in this manner have improved productivities of valuable metabolites and enzymes as well as retain their autotrophy and non‐GM status. Thus, they are considered suitable for use in the food industry.

**Figure 3:**
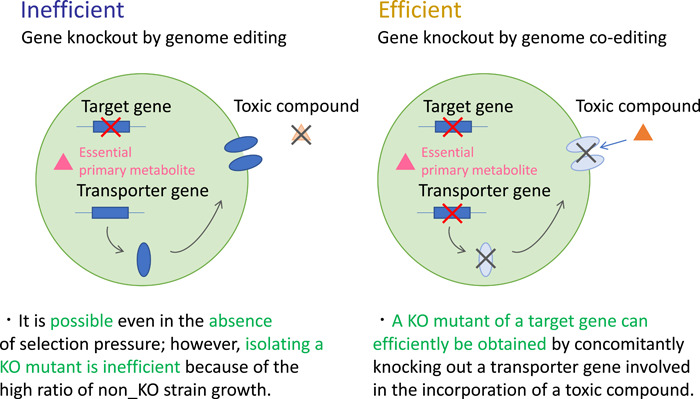
The mechanism by which genome co‐editing of target and membrane transporter genes works for the successful knockout of the target gene. No knockout (KO) mutant can be obtained efficiently if only the target gene is knocked out by genome editing (left panel). The mutant strain harboring KO of the target gene can be obtained if the target gene and a membrane transporter gene are concomitantly knocked out by genome co‐editing (right panel). In the case of a study on *wA* and *thiI* co‐editing (Todokoro et al., 2020), the generated mutant strain can grow normally as it can originally synthesize an essential primary metabolite thiamine de novo, while a toxic compound pyrithiamine that is an analog of the metabolite cannot be incorporated into cells due to loss of a responsible membrane transporter. Interruption of membrane transport does not become a problem in this case

Taken together, the key point of genome co‐editing for industrial fermentation use is that the target gene must be knocked out concomitantly with a membrane transporter gene. By so doing, we can specifically construct a non‐GM strain that is autotrophic and has a null mutation in the target gene (Figure 3). Currently, there is only one article on knocking out a target gene concomitantly with a membrane transporter gene *thiI* (Todokoro et al., 2020). Hereafter, if transporter genes whose knockout mutants can be selected by toxic compounds are additionally used, concomitant knockout of another target gene and another membrane transporter gene can be attained by genome co‐editing. By repeating genome co‐editing in this manner, autotrophic non‐GM mutants with multiple target gene knockouts could be created, along with knockout of multiple transporter genes. This technology can be crucial for breeding filamentous fungi in an industry‐tolerable form, as knockout of multiple target genes can largely enhance the productivity of a valuable metabolite or enzyme in a stepwise manner (Tamano & Yoshimi, 2021).

This study focuses on the advantages and prospects of genome co‐editing in creating improved strains of filamentous fungi. Moreover, this concept of concomitant knockout of a target gene and a membrane transporter gene seems applicable to other microorganisms, such as bacteria and yeasts. However, a prerequisite for this technology is that the microorganism must possess a membrane transporter gene whose knockout mutant can be selected with selection pressure. For example, the *sB* gene is considered to be involved in the transport of sulfate ions in *A. oryzae*, and selenate ion is reported to inhibit growth as a toxic analog of sulfate ion (Toyoshima et al., 2012; Yamada et al., 1997). Furthermore, the *nrtA* gene is reported to encode a membrane transporter of nitrate ion in *A. oryzae*, and chlorate ion is shown to inhibit growth as a toxic analog of the nitrate ion (Sano, 2016). Thus, it is desirable to firstly find such a transporter gene by literature review, sequence homology searches, reverse genetics experiments, and so on. I would recommend the application of genome co‐editing technology to create improved autotrophic non‐GM strains of filamentous fungi that are used in the food industry to produce valuable metabolites and enzymes.

## AUTHOR CONTRIBUTIONS

**Koichi Tamano**: Conceptualization (lead); data curation (lead); investigation (lead); project administration (lead); resources (lead); visualization (lead); writing—original draft (lead); writing—review & editing (lead).

## CONFLICT OF INTEREST

None declared.

## ETHICS STATEMENT

None required.

## Data Availability

All data are provided in this article.
